# Olverembatinib: A New Treatment for Adult Patients with Philadelphia Chromosome-Positive Acute Lymphoblastic Leukemia

**DOI:** 10.3390/cancers18121990

**Published:** 2026-06-18

**Authors:** Xavier Thomas

**Affiliations:** Hospices Civils de Lyon, Department of Clinical Hematology, Centre Hospitalier Universitaire, F-69003 Lyon, France; xavier12thomas@gmail.com

**Keywords:** acute lymphoblastic leukemia, tyrosine kinase inhibitors, Philadelphia chromosome, olverembatinib, treatment, *T315I* mutation, prognosis

## Abstract

Background/Objectives: The prognosis of patients with Philadelphia chromosome-positive (Ph^+^) acute lymphoblastic leukemia (ALL) has improved with the addition of tyrosine kinase inhibitors (TKIs) to conventional chemotherapy. However, there are limited therapeutic options for patients resistant or intolerant to current TKIs. Methods: A comprehensive search was made on PubMed.ncbi.nlm.nih.gov for published studies and ClinicalTrials.gov for registered trials, regarding the first results of and promising strategies with olverembatinib, a novel third-generation TKI, for the treatment of Ph^+^ ALL. Results: First trials involving olverembatinib showed significant anti-leukemic clinical activity both in newly diagnosed and relapsed/refractory patients with Ph^+^ ALL, especially those harboring the *T315I* mutation. Furthermore, olverembatinib demonstrated a favorable tolerability compared with the other TKIs. Conclusions: This augurs a new era in the standard of care for many patients with unmet clinical needs. However, further studies are warranted to assess olverembatinib’s real value and its cost-effectiveness.

## Simple Summary

The prognosis of patients with Philadelphia chromosome-positive acute lymphoblastic leukemia (Ph^+^ ALL) improved dramatically over the last decades with the introduction of tyrosine kinase inhibitors (TKIs). However, resistance or intolerance to TKIs remains a challenge. The novel third-generation TKI, olverembatinib, showed the potential to address unmet clinical needs in this situation. Fist clinical trials suggest impressive anti-leukemic activity, conferring to olverembatinib the potential to change to standard of care for patients with Ph^+^ ALL, especially those harboring an unfavorable mutation. The aim of this review is to summarize the first results obtained with olverembatinib and to discuss the potential therapeutic strategies using this novel agent for the treatment of Ph^+^ ALL.

## 1. Introduction

Olverembatinib, an oral, third-generation BCR::ABL1 tyrosine kinase inhibitor (TKI), also known as HQP1351 and GZD824, is currently being mainly investigated in the treatment of chronic myeloid leukemia (CML) and more recently Philadelphia chromosome-positive (Ph^+^) acute lymphoblastic leukemia (ALL). The drug, initially developed by Ascentage Pharma, has demonstrated promising anti-leukemic activity in heavily pre-treated patients. In November 2021, it was approved, at the dosage of 40 mg every other day (QOD), in China for the treatment of adult patients with TKI-resistant chronic- and accelerated-phase CML harboring the threonine-to-isoleucine mutation at position 315 (*T315I*), and in adult CML patients intolerant to first- and second-generation TKIs [1]. More recently, olverembatinib has been granted orphan drug designation and fast track designation by the US Food and Drug Administration (FDA) and orphan designation by the European Medicines Agency (EMA). Since 2024, the drug has been developed by Takeda in all territories outside of China, Taiwan and Russia.

This review provides a comprehensive overview of olverembatinib’s therapeutic potential and summarizes its first results in the treatment of Ph^+^ ALL.

## 2. Historical Considerations

ALL is a malignant hematological disease characterized by aberrations in proliferation and differentiation of lymphoblasts. Among adults with B-lineage ALL, Ph^+^ ALL characterized by the presence of a Philadelphia chromosome [t(9;22)] cytogenetic abnormality, resulting in the molecular juxtaposition of two genes, *BCR* and *ABL*, to form an aberrant *BCR::ABL* gene on chromosome 22, represents more than 25% of the cases. Historically, the prognosis of Ph^+^ ALL was considered very dismal, with a long-term survival rate of less than 10% after standard intensive chemotherapy [2,3], and could only reach 30 to 40% in patients able to undergo allogeneic stem cell transplantation (SCT) in first complete remission (CR) [4]. Prognosis improved substantially after 2000 with the addition of BCR::ABL1 TKIs to intensive chemotherapy, beginning with imatinib [5,6,7], and then second-generation TKIs dasatinib [8,9] and nilotinib [10]. A benefit was mainly demonstrated when TKI was started immediately, administered continuously, and continued indefinitely, leading, in combination with chemotherapy, to morphological CR rates of more than 90% and long-term survival rates reaching 50%. Given the efficacy of TKIs, some studies considered TKI-based regimens when combined with low-intensity chemotherapy [6,9,10] or no chemotherapy [11,12,13,14]. While a complete molecular response was observed in 20 to 50% of cases, the emergence of a *T315I* mutation was detected in up to 75% of patients relapsing after treatment with first- or second-generation TKIs [15]. Recently, ponatinib, a third-generation BCR::ABL1 TKI, combined with chemotherapy, showed complete molecular remission rate ranging from 71 to 86% and a long-term survival rate from 75 to 92% in newly diagnosed Ph^+^ ALL patients [16,17]. Its activity was demonstrated against both wild-type and mutated *ABL1* kinase domain including the *T315I* mutation. Superiority of ponatinib over first- or second-generation TKIs was confirmed in a randomized trial [18] and meta-analysis [19]. Therapeutic strategies also potentially moved to combinations with monoclonal antibodies targeting CD20 (rituximab) [20], CD19 (blinatumomab, a bispecific T-cell engager targeting CD3 and CD19) [13,21], or CD22 (inotuzumab ozogamicin, an antibody–drug conjugate) [22] in both the salvage and frontline settings. Recently, blinatumomab was combined with ponatinib in a chemotherapy-free regimen. This treatment resulted in a molecular remission rate of 85% and a 3-year survival rate of 90%. These results suggested a decreased need for allogeneic SCT in remission (historically considered the gold standard for all patients with Ph^+^ ALL), especially for those achieving measurable residual disease (MRD) negativity at a sensitivity of 10^−6^ [23]. Positive results were also obtained when blinatumomab was only combined with dasatinib [24]. Results of significant clinical studies obtained over the years for the treatment of Ph^+^ ALL are summarized in Table 1.

**Figure 1:**
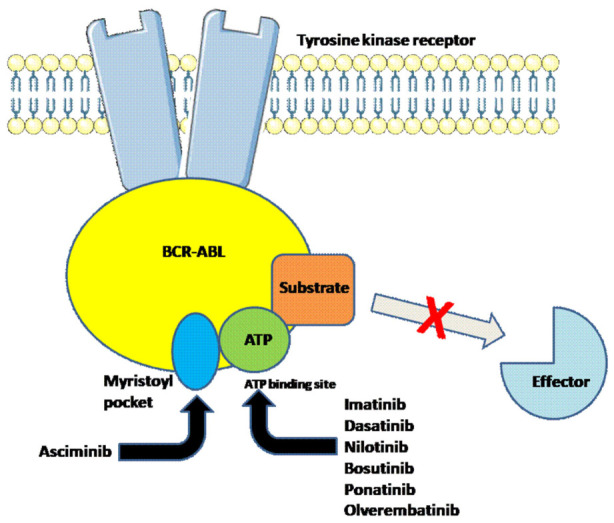
Mechanism of action of TKIs. Olverembatinib exerts its therapeutic effect by directly competing with ATP for the binding site within the catalytic domain of the BCR-ABL1 kinase. This competitive inhibition prevents the autophosphorylation and activation of the kinase, therefore blocking the downstream signaling pathways. Unlike olverembatinib and approved TKIs, asciminib inhibits the ABL1 kinase activity through binding to the ABL myristoyl pocket.

However, despite the impact of TKIs in the treatment of Ph^+^ ALL, significant unmet needs remain, especially for patients whose disease is resistant or refractory to these therapies or who develop mutations following these treatments. Besides *T315I* mutation, patients displaying additional *IKZF1* plus *CDKN2A/B* and/or *PAX5* deletions or *VPREB1* deletion were poor responders [25]. Introduction of novel therapeutic agents appears therefore required. In this setting, asciminib and olverembatinib have recently been developed. Asciminib inhibits the ABL1 kinase activity through binding to an allosteric site, the ABL myristoyl pocket, unlike the remaining BCR::ABL1 TKIs which bind to the ATP binding site of the BCR::ABL1 oncoprotein [26] (Figure 1). Olverembatinib is a pan BCR::ABL1 TKI which first trials demonstrated efficacy in Ph^+^ hemopathies previously treated with two or more TKIs, including hemopathies harboring severe mutations [1].

## 3. Olverembatinib: A New Third-Generation BCR::ABL1 TKI

**Figure 2:**
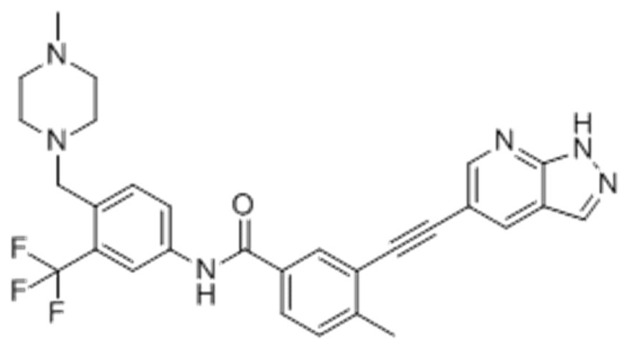
Chemical structure of olverembatinib.

Olverembatinib is an ATP binding-site inhibitor. It targets both wild-type BCR::ABL1 kinase and a broad spectrum of BCR::ABL1 mutants, including mutant *T315I*, known as the gatekeeper mutation, which confers resistance against all first- and second-generation TKIs [27,28]. The drug can also target other kinases, such as KIT, FLT3, FGFR1, and PDGFRα, potentially opening treatment opportunities for the management of acute myeloid leukemia or other malignancies, including gastro-intestinal stromal tumors (GISTs), endometrial cancer, and renal cell carcinoma [29]. Its molecular formula is C_29_H_27_F_3_N_6_O (Figure 2). In vitro, olverembatinib showed more activity than all other TKIs against BCR::ABL1-positive cells expressing various variants including *T315I* [30]. This includes ponatinib and asciminib. Comparisons with ponatinib point to differences in binding to the active and inactive conformations of wild-type and mutant BCR::ABL1 mutants [31]. Olverembatinib binds to both phosphorylated and non-phosphorylated conformations, while ponatinib binds only to non-phosphorylated wild-type and *T315I* mutant *ABL1*. Oral bioavailability of olverembatinib is compatible with alternate-day dosing and an appropriate half-life [27]. The absorption is slow with a mean terminal elimination half-life of 20 h. Olverembatinib is mainly metabolized by CYP3A4. Its administration should be cautious with strong CYP3A4 inhibitors or inducers, which may substantially alter olverembatinib pharmacokinetics and potentially exacerbate olverembatinib-induced toxic reactions. Coadministration of olverembatinib with itraconazole increases the maximum plasma concentration and duration of exposure of olverembatinib, while coadministration with rifampin decreases both its concentration and duration of exposure, which should drive to appropriate dose modifications if coadministration is required [32]. Pre-clinical studies showed inhibition of Ba/F3 murine cells expressing the native *BCR::ABL1* gene and resistance to mutations including *T315I* which contraindicates treatment with imatinib and second-generation TKIs, *V299L* and *F317L* mutations which contraindicate dasatinib and bosotinib, *Y253H*, *E255K/V*, and *F359V/C/L* mutations which contraindicate nilotinib, and *A337T*, *P465S*, *M244V*, and *F359V/I/C* which contraindicate asciminib [27]. Inhibition was then confirmed in mice xenografted with human ALL cells and on human CML cells. Olverembatinib was effective against Ba/F3 cells co-expressing *BCR::ABL1^T315I^* and luciferase, and increased lifespan of leukemic mice compared to controls [27]. Anti-leukemic effects of olverembatinib in Ba/F3 murine cells harboring *T315I* mutation were potentiated by combination with asciminib [33]. A potential impact of olverembatinib on leukemic blast clearance from the cerebrospinal fluid was suggested in an early clinical study involving relapsed patients with central nervous system (CNS) involvement [34]. Anti-leukemic effects of olverembatinib have also been demonstrated independently of BCR::ABL1 kinase, through inhibition of both PI3K/AKT and SRC kinase pathways [28].

## 4. Lessons from CML

The translocation of the *ABL1* gene from chromosome 9 to the *BCR* gene at its site on chromosome 22 creates the *BCR::ABL1* fusion gene, which transforms hematopoietic stem cells into leukemic cells through uncontrolled tyrosine kinase activity [35]. Ponatinib is currently the standard therapeutic option in CML patients harboring the *T315I* mutation. Major cytogenetic response (MCyR) and major molecular response (MMR) rates after 12 months of treatment reach 70% and 40% respectively [36]. Although harboring a different mechanism of action, asciminib demonstrated similar efficacy than ponatinib [37,38]. However, patients who are resistant or intolerant to ponatinib and asciminib need further therapeutic options. Olverembatinib, another third-generation TKI, appears potentially effective in this situation. It was first investigated in chronic-phase CML and accelerated-phase CML. A phase 1/2 study in CML patients with TKI resistance, of whom more than 60% harbored the *T315I* mutation, demonstrated olverembatinib efficacy [39]. Forty mg once every two days in 28-day cycles was established as the phase 2 dose, leading to drug approval in China with this schedule [40]. However, a real-world study suggested that an approach starting at a lower dose might allow similar efficacy with improved tolerability [41]. Patients starting at 30 mg once every two days experienced fewer dose reductions or treatment discontinuations, and maintained this initial dose through follow-up. This was confirmed by another study, which however showed that a higher concentration of olverembatinib was needed to inhibit leukemia cells with *T315I* mutation [42]. In chronic-phase CML patients, complete hematologic response (CHR), MCyR, complete cytogenetic response (CCyR), and MMR after 37 months of follow-up were 100%, 79%, 69% and 56%, respectively. They were 73%, 47%, 47% and 45%, respectively, in accelerated-phase CML. Grade ≥ 3 non-hematologic and hematologic treatment-related adverse events were seen in 49% and 56% of patients in chronic- and accelerated-phases, respectively. However, olverembatinib was well tolerated. Most treatment-related adverse events resolved over time. The most frequent non-hematologic side effects with olverembatinib therapy were skin hyperpigmentation manifested as lentiginous nevi, elevated creatine phosphokinase, hypocalcemia, and hypertriglyceridemia [39,43,44]. Arterial and venous occlusive events of special concern with ponatinib have yet to be investigated. Results after a longer duration of follow-up were consistent with the first analyses [43]. The phase 2 Chinese pivotal study (NCT04126681) randomized patients to a 2:1 ratio to either olverembatinib or investigator’s choice of best available treatment (but excluding bosutinib, ponatinib or asciminib) [45]. Olverembatinib significantly prolonged event-free survival (EFS) versus best available treatment (21 months versus 3 months) in the intention-to-treat population. Achieving cytogenetic and molecular responses over 36 months were also significantly higher with olverembatinib. An American study confirmed the results from the Chinese studies and demonstrated that a dosage of 30 mg was effective in patients without *T315I* mutation, while higher dosages were required in patients harboring the mutation [42]. Furthermore, this study found a strong anti-leukemic activity in chronic-phase CMLs known for resistance to ponatinib andasciminib [42]. Most cases of ponatinib and/or asciminib resistance that responded to olverembatinib had the *T315I* variant. Interesting results were also obtained by Chinese studies combining olverembatinib with venetoclax or hypomethylating agents in blastic-phase CML [46,47]. A study evaluating health-related quality of life (HRQoL) scores showed that being younger than 40 years and in chronic-phase CML was associated with greater improvements in HRQoL than being older and in accelerated-phase CML, as patients underwent more cycles of olverembatinib therapy [48].

## 5. Olverembatinib in Ph^+^ ALL

Ponatinib has shown efficacy in Ph^+^ ALL and currently represents the treatment of choice in frontline therapy or in relapsed/refractory Ph^+^ ALL after initial treatment involving first- or second-generation TKIs. Combined with chemotherapy regimens, CR rates reached 80 to 90% and 41% of relapsed/refractory Ph^+^ ALL achieved major hematologic responses [36]. However, ponatinib was not easily available in mainland China. This led, especially in China, to the development of olverembatinib in these indications. Combined with a pediatric-inspired chemotherapy protocol, olverembatinib used at 40 mg once every two days demonstrated increased MCyR achievement from 15% on day 14 to 85% on day 90 when compared to chemotherapy combined with another TKI [49]. Although tested on a small series, overall response rate (ORR) was 100% and CR was achieved regardless of the P190 or P210 transcript subtypes. Promising results were confirmed in further small series combining olverembatinib with standard or low-intensity chemotherapy in patients with newly diagnosed or relapsed/refractory Ph^+^ ALL [34,50,51,52]. High early and sustainable MCyR rates were achieved. Like in CML studies, most observed adverse events were mild and manageable. Common treatment-related adverse events included skin hyperpigmentation, hypertriglyceridemia, proteinuria, thrombocytopenia, and neutropenia. Various possible strategies have been proposed to manage myelosuppression during treatment, including low-intensity chemotherapy scheme, hematopoietic growth factor support, scheduled breaks in therapy, or dose reduction after achieving molecular response. Olverembatinib was also tested alone or combined with only vincristine and steroids in relapsed/refractory patients, including patients harboring *T315I* mutation and patients with prior treatment by ponatinib [53]. MCyR could be achieved in about 67% of cases and responses were obtained in some previously treated with ponatinib. In another study, 43% of patients resistant to ponatinib achieved MCyR, 29% achieved CCyR, and 22% achieved MMR after olverembatinib therapy [42]. Relapsing patients without *T315I* mutation obtained higher CCyR and MCyR rates than those harboring the mutation (33% versus 20% and 50% versus 20%, respectively). Olverembatinib was also incorporated within a regimen including high-intensity chemotherapy cycles and the bispecific T-cell engager (BiTE) antibody blinatumomab in patients with newly diagnosed Ph^+^ ALL [54]. MCyR rate was 35% after one cycle of treatment and reached 70% after another cycle. The one-year EFS and overall survival (OS) reached 100% at a median follow-up of one and half years. Beside combination with blinatumomab, first case reports revealed a complementary action when combining olverembatinib with the anti-CD22 antibody, inotuzumab ozogamicin [55]. Similar results were obtained with the combination of olverembatinib, venetoclax and dexamethasone (OVD regimen) for induction and consolidation phases with a course of treatment every 28 days (NCT06082934) [56]. In this trial, olverembatinib was used at 40 mg orally every other day, along with intravenous dexamethasone (10 mg on days 1 to 14 and 5 mg on days 15 to 28). Venetoclax was administered at 100 mg on day 4, 200 mg on day 5, and 400 mg on days 6 to 17. After CR achievement, olverembatinib was administered at 40 mg every other day on days 1 to 28 and venetoclax 400 mg daily for the first two weeks of each cycle. All patients achieved CR and MRD-negativity, and 80% of them obtained MMR. Favorable outcomes with olverembatinib and venetoclax-based regimen were confirmed by other studies suggesting complementary mechanisms of action [57]. Responses to olverembatinib therapy were noted not only in relapsed patients harboring the *T315I* mutation [55], but also for patients harboring other mutations, such as the *E255V* mutation [58]. Main registered current studies involving olverembatinib in the treatment of Ph^+^ ALL are summarized in Table 2. Most of them tend to test olverembatinib in frontline therapy. The position of olverembatinib within regimens was evaluated in induction and consolidation phases, but also as maintenance therapy including the post-transplant phase (NCT07074496, NCT05466175, NCT06658925). Maintenance therapy with TKIs after allogeneic HSCT in Ph^+^ ALL can decrease relapse incidence and improve OS. It was shown that the efficacy of this maintenance therapy was better was started within the three months after transplantation [59]. In a recent retrospective study using olverembatinib alone as maintenance post-transplant, the 3-year OS and relapse-free survival (RFS) was 92% and 79%, respectively [60]. The efficacy of olverembatibib as maintenance therapy after transplantation was similar to that reported with ponatinib and appeared well tolerated [61]. Combinations mostly involve low-intensity chemotherapy (NCT07152041, NCT07493161), including inhibitors of BCL2 (NCT05495035, NCT06481228, NCT06754267, NCT06082934, NCT05594784) and monoclonal antibodies (NCT05931757, NCT07178912, NCT06220487, NCT07443488). Two phase 2 studies combining olverembatinib with inotuzumab ozogamicin as a bridging therapy prior to transplantation recently conducted enrolling patients with persistent MRD (NCT05603156) after at least three rounds of chemotherapy, and patients with relapsed/refractory Ph^+^ ALL (ChiCTR2200061432). Treatment resulted in a high complete molecular response (CMR) rate, a high bridging transplantation rate, and a good tolerance [62]. Another proposed study takes into account genetic alterations (NCT07454226). Patients with ABL class fusions (*ABL1*, *ABL2*, *PDGFRA*, *PDGFRB*) receive olverembatinib, while those with JAK pathway alterations (*CRLF2* rearrangement, *JAK* mutation/fusion, *EPOR* fusion, *SH2B3* deletion, *IL7R* mutation) receive gecacitinib (also known as jaktinib), a novel inhibitor targeting both JAK and ACVR1.

## 6. Discussion

The novel third-generation TKI, olverembatinib, appears well tolerated and efficacious both in newly diagnosed and heavily pre-treated TKI-resistant patients with Ph^+^ ALL. Olverembatinib exerts sustainable anti-leukemic activity in patients including those with the *T315I* mutation and other mutations conferring resistance to other TKIs. In newly diagnosed patients with Ph^+^ ALL, olverembatinib combined with low-intensity chemotherapy gave 64% of MRD-negativity by the end of induction, including patients with unfavorable prognostic genotypes (POLARIS 1 study, NCT06051409) [63]. Furthermore, olverembatinib tolerability compared favorably with the other TKIs (Table 3). The most common adverse event was skin hyperpigmentation, which was not noted with other TKIs. The precise mechanism of this adverse event is not known and should be elucidated, but could be explained by the inhibition of various kinases. Severe thrombocytopenia is another event that appeared more frequent than what was observed with ponatinib, but severe cardiovascular events were less frequent than those reported in ponatinib studies [36]. Molecular features, including the presence of *ASXL1*, *FAT4* and/or *TET2* mutations, were recently identified as independent predictors for glucolipid metabolic disorders and cerebrovascular adverse events, and therefore guide early risk identification and targeted surveillance [64]. Although effectiveness of olverembatinib was comparable to that observed with ponatinib and asciminib in newly diagnosed patients [36,65], very encouraging effects were noted in patients previously treated with those agents, suggesting especially a higher specific targeting of the *T315I* mutation with olverembatinib [42,53]. Overall, olverembatinib good tolerance combined with its profile as multikinase inhibitor suggests a potential utility in the treatment of this disease.

Despite those encouraging results, some issues should be mentioned. First, which patients will benefit from the introduction of olverembatinib has yet to be defined. Should it be given to all patients with Ph^+^ ALL or reserved for those previously heavily treated and having developed resistance or intolerance to prior therapy. In the first situation olverembatinib would become a new standard of care in this disease. In ALL, it is effectively generally admitted to use agents with the larger spectrum of activity from frontline therapy in order to avoid resistance or relapse always harder to eradicate. Persistence or reappearance of MRD is the most important adverse prognostic factor in ALL. Combinations yielded deeper responses and remissions are therefore required to allow organization of allogeneic SCT, which remains the main goal of therapy in high-risk patients, and to perform it in the best conditions. In the later situation, olverembatinib would be limited to salvage therapy. Patients who are resistant to ponatinib or to asciminib need further therapeutic options [66] and olverembatinib has demonstrated a potential efficacy in this setting [42]. The mechanism that could explain the superiority of olverembatinib over ponatinib remains unknown. However, olverembatinib can bind to both non-phosphorylated and phosphorylated BCR::ABL1, while ponatinib only bind to non-phosphorylated BCR::ABL1 [27,31]. Theoretically, therapies with an alternative mechanism of action are considered the best choice in case of relapse or resistance. Unlike ABL-TKIs, which target the ATP binding site, asciminib is a novel drug that shows a therapeutic effect by binding to the myristoyl pocket, which regulates ABL activity [65]. However, asciminib did not demonstrate any superiority comparatively to other novel inhibitors. Asciminib showed similar effects than the third-generation TKI ponatinib [37,38], but the progression-free survival (PFS) was lower for ponatinib pre-treated patients than for ponatinib naïve patients [67,68]. Regarding olverembatinib, efficacy was noted in some cases pre-treated by asciminib, while no cases were reported until now in the opposite situation.

**Figure 3:**
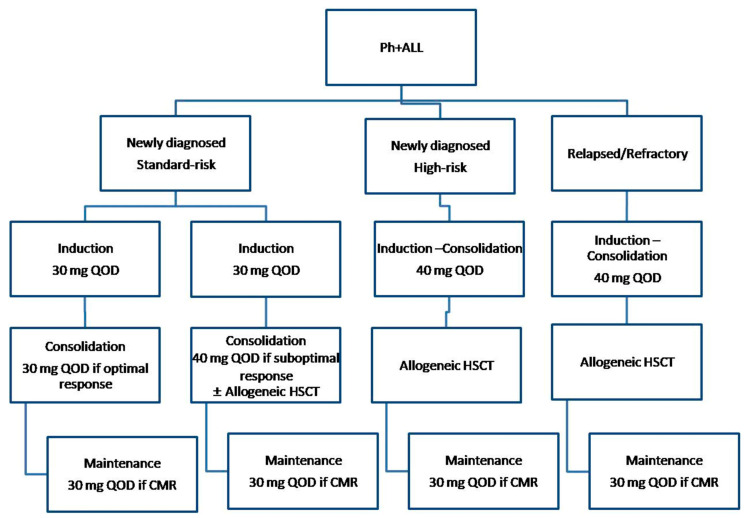
Algorithm regarding olverembatinib dosing according to Ph^+^ ALL status.

Second, which dosing should be used? Clinical experience already underscores the importance of flexible TKI dosing [30]. Olverembatinib 30 mg QOD as a starting dose demonstrated equivalent efficacy and better tolerability compared with 40 mg QOD. Starting olverembatinib at a lower dose with the flexibility to escalate based on suboptimal response seems therefore consistent with real-world practice patterns [41]. Maintaining a good quality of life and adherence to treatment appears as important as achieving deep molecular responses. However, 40 mg QOD demonstrated a better efficacy in patients harboring an unfavorable mutation. A tentative of algorithm regarding olverembatinib dosing that should be used is presented in Figure 3.

Third, questions remain about how best to use olverembatinib: as a single agent or in combination therapy, and what combination therapy, for induction, consolidation, and/or maintenance, or as part of a large combination or succession of treatments. Administration should certainly follow the same rules as other TKIs. Treatment should be started immediately and administered continuously for induction and consolidations rather than intermittently, and continued indefinitely. Although intensive chemotherapy remains still required in Ph^+^ ALL with *IKZF1* deletion plus additional genetic aberrations including *CDKN2A*, *CDKN2B*, or *PAX5*, an important current point in the treatment of Ph^+^ ALL is the transition of patients to a chemotherapy-free era. Recently blinatumomab and venetoclax were integrated into chemotherapy-free regimens with TKIs serving as the backbone in the treatment of Ph^+^ ALL. A combination of blinatumomab with TKIs already demonstrated interesting results with a MCyR rate of 75% and a one-year OS rate of 73% in relapsed/refractory patients [69]. In the frontline setting, the combination of ponatinib with blinatumomab showed 46% to 59% of MCyR after induction [23,70] and 96% of MRD-negativity [23]. The 3-year EFS and OS rates were 78% and 88%, respectively [71]. In this setting, olverembatinib combined with blinatumomab already showed promising results [54]. Several ongoing trials should confirm these preliminary results (NCT05931757, NCT07178912). Olverembatinib combined with venetoclax and dexamethasone is another current attractive chemotherapy-free regimen (NCT06082934). Addition of venetoclax or another *BCL2* inhibitor, which exhibits a high activity in relapsed/refractory ALL [72], completes effectively achievement of rapid and profound remissions. The first assessment of the OVD regimen in frontline therapy showed achievement of MRD-negativity in all patients at day 14 and CMR in 90% of them at day 90 that were maintained throughout the follow-up period [56]. The effectiveness and safety of olverembatinib combined with blinatumomab and venetoclax is currently evaluated in several ongoing trials.

Finally, the ability to target sanctuary sites remains, like for other TKIs, another major challenge. Primary results of the NCT05495035 clinical trial combining olverembatinib with APG-2575 (lisaftoclax) showed that both agents were detectable in the cerebrospinal fluid indicating CNS penetration, positioning this combination as a highly promising strategy in Ph^+^ ALL with CNS involvement [73]. Besides the increased efficacy of novel TKIs, other ways are currently explored to overcome TKI resistance in Ph^+^ ALL. In this setting thermal-based therapy was recently presented as offering a promising adjuvant approach [74]. Heat stress was shown to suppress proliferation of *BCR::ABL1*-driven leukemic cells, including drug resistant mutant in vitro and decrease tumor burden in vivo.

## 7. Conclusions

Overall, first clinical trials with overembatinib suggest impressive anti-leukemic activity in patients with Ph^+^ ALL. Olverembatinib has the potential to change the standard of care for adults with Ph^+^ ALL, and could hold in combination therapy the promise of substantial benefit, especially in patients harboring an unfavorable mutation. Larger series and longer follow-up intervals are warranted to confirm olverembatinib value, further assess its toxicity profile, especially in the cardiovascular setting, and its cost-effectiveness, an essential consideration in resource-limited and/or low-income countries [75].

## Figures and Tables

**Table 1:** Examples of results from clinical studies reflecting the evolution of treatment and prognosis over the years for Ph^+^ ALL (from the 80s to the present).

Treatment	Status	Results	Ref.
**Chemo ± HSCT**	ND	CR: 64%; Med DFS: 6 m; Med OS: 9 m	[3]
	ND	CR: 62%; 3 y-OS: 19%; MRD-neg: 38%; 3 y-OS(Allo): 28%	[4]
**Imatinib + Chemo ± HSCT**	ND	CR: 93%; MRD-neg: 88%; 5 y-DFS: 43%; 5 y-OS: 43%	[5]
	ND	CR: 91%; MMR: 64%; 5 y-EFS: 32%; 5 y-OS: 43%	[6]
	ND	CR: 92%; 4 y-OS: 38%	[7]
**Second-gen TKI + Chemo ± HSCT**	ND	CR: 88%; 3y-EFS: 55%; 3 y-OS: 69%; 3 y-RFS: 62%; 1 y-RFS (post-transpl): 71%; 1 y-OS (post-transpl): 87%	[8]
	ND	CR: 97%; MMR: 71%; 4 y-OS: 79%; 4 y-RFS: 76%	[10]
**TKI + reduced-intensity chemo**	ND	CR: 98%; MMR: 66%; 5 y-EFS: 42%; 5 y-OS: 48%	[6]
	ND old	CR: 96%; MRD < 10^−3^: 65%	[9]
	ND	CR: 99%; MMR: 77%; 4 y-OS: 73%; 4 y-RFS: 59%	[10]
**TKI (Chemo-free)**	ND old	CR: 100%; Med MRD reduction: 2.0–2.9	[11]
	ND	CR: 100%; MRD < 10^−3^: 19%; 1.5 y-DFS: 51%; 1.5 y-OS: 69%	[12]
	ND old	CR: 86%; CMR: 41%; Med EFS: 14.3 m; Med OS: NR	[14]
**Ponatinib + chemo ± HSCT**	ND	CR: 100%; CMR: 86%; 6 y-EFS: 65%; 6 y-OS: 75%	[16]
	ND	CMR (post-ind): 47%; CMR (post-conso): 71%; 2 y-EFS: 70%; 2 y-OS: 96%	[17]
**Introduction of Blina**	R/R	CR: 36%; CMR: 31%; Med RFS: 6.8 m; Med OS: 9 m; Dur CMR: 9.7 m	[21]
**Dasa + Blina (Chemo-free)**	ND	4.5 y-DFS: 76%; 4.5 y-OS: 81%; 4.5 y-EFS: 75%	[13]
**Pona + Blina (Chemo-free)**	ND + R/R	CMR: 87%; CMR (R/R): 79%	[23]

Abbreviations: Allo, allogeneic transplantation; Blina, blinatumomab; Chemo, chemotherapy; CMR, complete molecular response; CR, complete remission; conso, consolidation; Dasa, dasatinib; DFS, disease-free survival; Dur, duration; EFS, event-free survival; HSCT, hematopoietic stem cell transplantation; ind, induction; m, month; Med, median; MMR, major molecular response; MRD, measurable residual disease; ND, newly diagnosed; neg, negative; old, older patients; OS, overall survival; Pona, ponatinib; RFS, relapse-free survival; R/R, relapsed/refractory; second-gen, second-generation; transpl, transplantation; TKI, tyrosine kinase inhibitor; y, year.

**Table 2:** Current registered treatments involving olverembatinib in Ph^+^ ALL.

Registration Number (Sponsor) Status	Indication (Phase) Treatment	Main Outcome Measures
**NCT05495035** (Institute of Hematology and Blood Diseases Hospital) Recruiting	R/R pediatric Ph^+^ ALL (Phase 1b) Olv, APG-2575	DLT, ORR, maximum plasma concentration, AUC
**NCT07152041** (Institute of Hematology and Blood Diseases Hospital) Recruiting	ND pediatric Ph^+^ ALL (Phase 3) Olv, Blina, Ven	d19 MRD, d48 end-of-induction MRD, EFS, OS, cumulative incidence of relapse
**NCT06481228** (Institure of Hematology and Blood Diseases Hospital) Recruiting	Adult Ph^+^ ALL (NA) Olv, Ven, reduced intensity chemo followed by CAR T-cell therapy as consolidation	Reduction in cycles of chemo, shortened length of hospitalization, improved QoL
**NCT06754267** (First Affiliated Hospital of Zheijiang University) Recruiting	ND adult Ph^+^ ALL (Phase 2) Olv, Ven, Prednisone	Safety and efficacy
**NCT07074496** (First Affiliated Hospital of Soochow University—Fundamenta Therapeutics, Ltd.) Active not recruiting	ND Ph^+^ ALL (Early phase 1) Chemo followed by ThisCAR19A therapy, Olv as maintenance	Efficacy, safety and PK of CAR T combined with Olv
**NCT05931757** (First Affiliated Hospital of Soochow University) Recruiting	ND adult Ph^+^ ALL (Phase 2) Olv, Blina	Efficacy and safety
**NCT06082934** (Xijing Hospital) Recruiting	ND Ph^+^ ALL (Phase 1/2) Olv, Ven, Dex	CR, MRD, MCyR, PFS
**NCT05594784** (Institute of Hematology & Blood Diseases Hospital) Completed	ND Ph^+^ ALL (Phase 2) Olv, Ven, reduced-intensity chemo	Efficacy and safety
**NCT07178912** (MD Anderson Cancer Center) Not yet recruiting	ND and R/R Ph^+^ ALL (Phase 2) Olv, sc Blina	ND: MCyR R/R: ORR
**NCT05466175** (Chen Suning) Not yet recruiting	ND Ph^+^ ALL (Phase 2) Olv, Chemo followed by cycles of Hyper-CVAD, a maintenance with Olv	Efficacy and safety
**NCT06051409** (Ascentage Pharma Group Inc.) Recruiting	ND Ph^+^ ALL (Phase 3) Olv, chemo vs. Imatinib, chemo	Randomization Efficacy and safety
**NCT07454226** (Institute of Hematology & Blood Diseases Hospital) Recruiting	ND Ph^+^ ALL (Phase 2) Olv or Gecacitinib according to ABL class fusions, Chemo, Ven	Efficacy and safety MRD-neg at 3 months
**NCT06220487** (Nanfang Hospital, Southern Medical University) Recruiting	ND Ph^+^ ALL (Phase 2) Olv, Blina, Chidamide	MCyR at 3 months, OS, EFS, AE, IKZF1/CD20 subgroups
**NCT07443488** (Institute of Hematology & Blood Diseases Hospital) Not yet recruiting	Ph^+^ ALL not achieving MRD-neg after induction chemo (Phase 2) Olv, Ino as first-line consolidation	MRD clearance rate
**NCT06658925** (Institute of Hematology & Blood Diseases Hospital) Not yet recruiting	Ph^+^ ALL treated by Allo HSCT (Phase 2) Olv as post-transplant maintenance	Prevention of recurrence
**NCT07493161** (Institute of Hematology & Blood Diseases Hospital) Recruiting	ND Ph^+^ ALL (NA) Olv, Ven, Blina, low-intensity chemo	Rate of *BCR::ABL1* ≤ 0.01% at d90, EFS
**NCT04260022** (Ascentage Pharma Group Inc.) Recruiting	Refractory Ph^+^ ALL and CML (phase 1b) Olv	PK, safety and efficacy
**NCT05603156** (Institute of Hematology and Blood Diseases Hospital) Recruiting	Ph^+^ ALL with MRD positive (NA) Olv, Ino	Efficacy and safety for MRD clearance before HSCT

Abbreviations: AE, adverse events; Allo HSCT, allogeneic hematopoietic stem cell transplantation; AUC, aera under the plasma concentration versus time curve; Blina, blinatumomab; chemo, chemotherapy; CAR T, chimeric antigenic receptor T; CR, complete remission; d, day; Dex, dexamethasone; DLT, dose-limiting toxicity; EFS, event-free survival; Hyper-CVAD, chemotherapy combining cycles of cyclophosphamide, vincristine, adriamycin, dexamethasone followed by cycles of methotrexate and cytarabine; Ino, inotuzumab ozogamicin; MCyR, major cytogenetic response; MRD, measurable residual disease; NA, not applicable; ND, newly diagnosed; neg, negativity; Olv, olverembatinib; ORR, overall response rate; OS, overall survival; PFS, progression-free survival; Ph^+^ ALL, Philadlphia chromosome-positive acute lymphoblastic leukemia; PK, pharmacokinetics; QoL, quality of life; R/R, relapsed/refractory; sc, sub-cutaneous; Ven, venetoclax.

**Table 3:** Comparison of olverembatinib with ponatinib and asciminib in newly diagnosed Ph^+^ ALL.

Features	Olverembatinib	Ponatinib	Asciminib
**Examples of responses in combination with reduced intensity treatment [Ref]**	CR: 100% MCyR: 85% [54]	ORR: 94% MRD-neg: 34% [18]	MRD-neg: 89% MMR: 74% [26]
**Main adverse events**	Skin hyperpigmentation Cytopenias Hypertriglyceridemia	Pancreatitis Arterial occlusive events Venous occlusive events Cytopenias	Arthralgias Cytopenias Pancreatitis
**Dosing**	40 mg QOD 30 mg QOD in newly diagnosed patients or in case of good molecular response	30–45 mg QD 15–30 mg QD if good molecular response	80 mg QD or 40 mg every 12 h 200 mg every 12 h if *T315I* mutation

Abbreviations: CR, complete remission; MCyR, major cytogenetic response; MMR, major molecular response; MRD-neg, measurable residual disease negativity; ORR, overall response rate; QD, daily; QOD, once every two days; Ref, reference.

## Data Availability

A comprehensive search was made on PubMed.ncbi.nlm.nih.gov for published studies and ClinicalTrials.gov for registered trials.

## Abbreviations

The following abbreviations are used in this manuscript:

ALL	Acute lymphoblastic leukemia
BiTE	Bispecific T-cell engager
CCyR	Complete cytogenetic response
CHR	Complete hematologic response
CML	Chronic myeloid leukemia
CMR	Complete molecular response
CNS	Central nervous system
CR	Complete remission
EFS	Event-free survival
EMA	European Medicines Agency
FDA	Food and Drug Administration
GIST	Gastro-intestinal stromal tumor
HRQoL	Health-related quality of life
MCyR	Major cytogenetic response
MMR	Major molecular response
MRD	Measurable residual disease
ORR	Overall response rate
OS	Overall survival
Ph^+^	Philadelphia chromosome-positive
QOD	Every other day
RFS	Relapse-free survival
SCT	Stem cell transplantation
TKI	Tyrosine kinase inhibitor
